# Triple Plane Dissection in Open Primary Rhinoplasty in Middle Eastern Noses

**Published:** 2013-08-05

**Authors:** Ahmed Elshahat

**Affiliations:** Plastic Surgery Department, Faculty of Medicine, Ain Shams University; and Eldemerdash Hospital, Cairo, Egypt

## Abstract

**Objective:** Rhinoplasty started as a closed technique and then the open technique gained popularity. Open technique gave surgeons the opportunity to visualize and manipulate the cartilaginous skeleton at the tip of the nose precisely. The dissection planes in open rhinoplasty technique may be subcutaneous, submuscular (under the superficial musculoaponeurotic system), or subpericondrial subperiosteal. Each plane has advantages and disadvantages. The aim of this study was to combine planes to get the maximal benefit of each plane. **Method:** The study was performed on 38 Middle Eastern patients, among whom 23 were females and 15 were males. All patients presented for primary rhinoplasty. They were divided into 5 groups on the basis of their skin thickness. Dissection started subcutaneous at the area of the lower lateral cartilages and then shifted subsuperficial musculoaponeurotic system over the upper lateral cartilages and ended subperiosteal over the bony skeleton. **Results:** This triple plane of dissection gave acceptable results without any complication. Subcutaneous dissection allowed thinning of the thick sebaceous skin at the tip and alar region, subsuperficial musculoaponeurotic system dissection allowed direct exposure of the upper lateral cartilage without thinning skin at an area where it is thin, and subperiosteal dissection helped masking any bony irregularities resulted from osteotomies. **Conclusion:** The triple plane dissection in open primary rhinoplasty in Middle Eastern patients maximized the advantages of each plane and minimized the disadvantages and resulted in safe cosmetic results.

In classic primary rhinoplasty techniques, whether they were closed or open, the dissection is a subsuperficial musculoaponeurotic system (sub-SMAS) (supraperichondrial and supraperiosteal), and then the periosteum is sharply incised in the midline and elevated with a Joseph periosteal elevator to the radix area and laterally to the extent necessary to allow bony hump reduction.^1^^,^^2^

Cakir et al^3^ adopted a complete subperichondrial subperiosteal dissection technique for rhinoplasty. They mentioned that a complete subperichondrial subperiosteal dissection technique resulted in relatively limited edema and more rapid patient recovery compared with their previous experience with the SMAS approach. Repeat elevation in the subperichondrial plane was easier and less traumatic in revision cases.

On the contrary, Safe and Sadek^4^ used the subcutaneous plane. They mentioned that the subcutaneous plane is the most convenient plane of dissection. Nasal muscles (nasal SMAS) are preserved, which guarantee animated nose postoperatively. The nasal SMAS is preserved in the Cakir et al^3^ study too, but the difference was that Safe and Sadek^4^ technique can readjust the tension of the muscle after reduction of the nasal skeleton which is not the case in Cakir et al^3^ technique.

Middle Eastern noses are characterized by heavy thick skin envelope, which together with the weak alar cartilage and short middle and medial crura contribute to the bulbous tip deformity with poor definition.^5^

Burget^6^ divided nasal skin into 3 zones on the basis of skin thickness and used this classification in decision making in esthetic reconstruction of the nose. Zone I is the thin loose nonsebaceous skin located over the nasal bone and upper lateral cartilages. Zone II is the thick sebaceous skin covering the alar cartilages and forming the tip. Zone III is the thin skin covering the columella and alar rims at the nasal base. Therefore, the heavy thick sebaceous skin is concentrated over the lower lateral cartilages and the tip. Daniel^5^ performed defatting of thick skin in 36% of his Middle Eastern patient.

Defatting of the thick sebaceous skin of the Middle Eastern noses is better achieved by dissection at the subcutaneous plane. Dissection at a deeper plane (sub-SMAS plane) would violate nasal muscles if defatting was attempted. This surely affects both function and esthetics adversely.

On the contrary, using the subcutaneous dissection plane at zone I may damage the skin and induce a long-lasting ecchymosis. This is the zone where the dorsal splint is applied postoperatively and minimal pressure may damage the skin if subcutaneous plane was used. It is better to use deeper planes for dissection at this zone.

Over the upper lateral cartilages, nasal SMAS is better elevated with the skin to expose the perichondrium. Over the nasal bone, the periosteum is better elevated with the overlying nasal SMAS and skin to expose the bone directly in 1 step to be able to rasp or osteotomize it according to the need.

Composite skin nasal SMAS will mask any irregularities in the osteocartilaginous skeleton. The periosteum protects the underlying bone from soft tissue invasion and enhances bone healing.^7^

The aim of this study was to present the idea of the triple plane dissection in open primary rhinoplasty in Middle Eastern patients. Combining planes provides the maximal benefit of each plane.

## Patients and methods

The study was performed on 38 patients, among whom 23 were females and 15 were males. All patients presented for primary rhinoplasty. Any patient who had any previous nose surgery was excluded. Patients with cleft lip nose deformity were excluded too. Ages ranged from 18 to 45 years.

History taking was mandatory in all patients. Of special importance in the history were the history of trauma, history of medications that may increase bleeding tendencies, and the presence of nasal airway obstruction.

Consent form was signed by every patient including approval to perform surgery and approval of taking photos and the use of these photos in teaching, presentations, and publications.

Preoperatively, all patients sustained clinical examination for assessment and planning. Evaluation of the thickness and sebaceous character of the nasal skin was the routine in all patients. Nasal deformities included different combinations of deviated nose, thick sebaceous skin, dorsal nasal hump, wide dorsum, bulbous ill-defined nasal tip, broad nasal base, hanging columella, alar flaring, and abnormal nasolabial or nasofrontal angles. Full preoperative laboratory investigations were done.

On the basis of N +3 to −3 system previously used by Daniel,^5^ the 38 patients were divided into 5 groups (Table 1). Management of skin in each group differed (Table 1).

Medical photography was taken for all patients in 6 positions: front view, base view, right oblique, right lateral, left oblique, and left lateral.

All operations were performed under general endotracheal intubation. The endotracheal tube was fixed on the central part of the lower lip. Pack was inserted in the throat.

Sterilization using Povidine iodine was done, and sterilized towels were used exposing the face including the auricles and chin. Anesthesiologist was instructed to maintain low blood pressure all over the surgery, and the head of the table was tilted 30º up. The surgeon stood on the right side of the head of the patient and the assistant surgeon on the left side.

### Surgical technique

Open rhinoplasty technique was used in all patients. Adrenaline saline 1/200000 was infiltrated into the planed planes of dissections to maintain bloodless field during surgery. Areas infiltrated included the columella in a subcutaneous plane, nasal septum in the submucoperichondrial plane bilaterally, nasal tip and the dorsum over the alar cartilages in a subcutaneous plane, and remaining dorsum and side walls in a deeper plane just superficial to the perichondrium of the upper lateral cartilages and deeper to the periosteum of the nasal bone.

Transcolumellar stair-step incision joined infracartilaginous incisions to open the nose. Dissection over the alar cartilage was performed in a subcutaneous plane. This left the alar cartilages covered by the nasal SMAS. Defatting the under surface of the thick skin was performed in indicated patients at this time. Plane of dissection is then shifted to the sub-SMAS plane at the junction of the thick skin of zone II and thin skin of zone I to expose the dorsal cartilaginous hump. Once the nasal bone was reached, the plane of dissection became subperiosteal.

**Figure 1:**
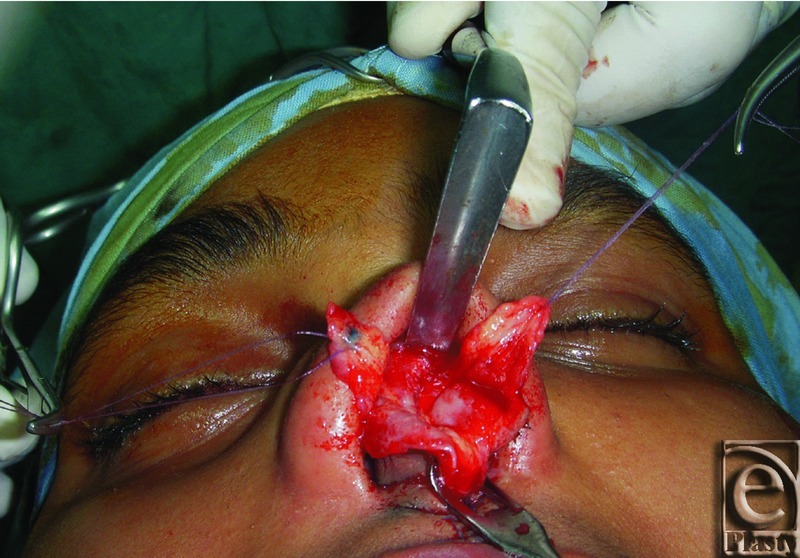
An intraoperative photograph shows elevation of the nasal SMAS as a separate layer from the skin and the alar cartilages.

Midline incision at the SMAS layer covering the alar cartilages was then done, and the SMAS layer dissected laterally from the perichondrium of the alar cartilages and left attached to the SMAS covering the sidewalls of the upper lateral cartilages (Fig 1). Exposure of the nasal septum was then done through the elevation of mucoperichondrial and mucoperiosteal flaps bilaterally, and the mucoperichondrial lining of the upper lateral cartilage was elevated for a short distance. Extramucosal hump excision was performed at this stage. The central septum proper was reduced first before any upper lateral cartilage resection.

Bony hump was managed either by rasps for small dorsal humps or by osteotome for large dorsal humps. If the hump did not reach the radix, bilateral medial osteotomies were performed. To close the open roof deformity, lateral percutaneous osteotomy using 2-mm osteotome was done to close the open dorsum. Finger pressure for 5 minutes after each lateral osteotomy was mandatory to lower the incidence of the postoperative ecchymosis. Redraping of the skin over the dorsum was done to check any irregularities that needed refinement.

Reduction of major dorsal hump necessitated the use of spreader grafts, which were harvested from the septal cartilages to prevent narrowing the internal nasal valves. Before harvesting the septal cartilage the caudal septum was shortened in cases of long noses and then the grafts were harvested leaving an L-shaped septum undamaged. This was followed by cephalic trim of alar cartilages and tip sutures or grafts according to the pathology.

Nasal SMAS layer was resutured after excising excess SMAS to regain muscle tension and the skin was redraped. The infracartilaginous incisions and columellar incisions were closed. Septal sutures to close the dead space between the 2 septal mucoperichondrial flaps were used. Nasal packs were applied and dorsal splint was placed carefully.

Throat pack was removed after suction of pooled blood and secretions. Once the patient was extubated, he or she was instructed to breathe through the mouth. Cold fomentations were applied and the patient was positioned in a semi-setting position. Nasal packs were removed after 48 hours and dorsal splint after 1 week. Columellar sutures were removed after 1 week. Patients were instructed to avoid wearing eyeglasses or sunglasses for 1 month.

## Results

Intraoperatively the triple plane dissection was easy to perform with no extra operative time needed. This allowed defatting of the thick skin over the alar cartilages and tip. Camouflaged any irregularities left after excision of the dorsal hump and osteotomies. No ecchymosis was noticed over the thin skin zone.

Postoperatively, all patient recovered from anesthesia and the postoperative period was uneventful. Minimal ecchymosis under the eyes and slight edema were noticed and resolved completely in less than a week. Nasal functions and mobility were maintained because nasal valves and nasal SMAS were preserved. Patients were followed up for 1 year.

Extensive defatting in the 5 patients with N +3 skin improved the skin quality but the esthetic tip was not achieved. The skin became thinner and less sebaceous. The patients accepted the limitation of the esthetic results because they were informed beforehand of the difficulty of their noses.

Defatting in the N + 2 group improved skin quality and allowed the achievement of esthetic tip. Results achieved in N, N + 1 and N − 1 groups was not due to modification of the skin thickness but rather due to modifications of the underlying cartilaginous skeleton.

Patients’ satisfactions were measured by asking the patient to rate the results as poor, good, very good, or excellent. But, this assessment carried a bias regarding the aim of this current study because the patients commented on the procedure as a whole.

Neither of the patients needed revision. Pre- and postoperative photographs for 5 patients in different photography positions for the noses are shown (Figs 2–31).

## Discussion

Middle Eastern noses are characterized by thick sebaceous skin especially at zone II of nasal skin thickness. Traditional sub-SMAS dissection plane makes defatting difficult and excision may involve removal of nasal SMAS at this zone, which affects dynamics at the tip of the nose. Guyuron^8^ mentioned that nasal muscle dysfunction is a hallmark of rhinoplasties performed 3 to 4 decades ago when the thin nasal muscles were damaged during dissection. Normally equilibrium of the dorsum tip of the nose is maintained by the antagonistic actions of the levator septi nasi muscle and the depressor septi nasi.^9^

Using the Cakir et al's^3^ complete subperichondrial dissection technique for rhinoplasty will not allow defatting the skin at the thick bulbous tip. Cakir et al's^3^ technique cannot fit the Middle Eastern noses with the thick sebaceous skin. The technique of subcutaneous dissection of Safe and Sadek^4^ allow defatting the thick skin and at the same time preserve the nasal SMAS. This technique was used in this study at zone II (thick skin zone).

The second plane of dissection in this study was the sub-SMAS plane at the region of cartilaginous hump. This differed from Cakir et al^3^ plane and was easier because there was no need to elevate perichondrium from the hump that was excised. This would have been time consuming with no benefit. At this region, sub-SMAS plane was better than subcutaneous plane of Safe and Sadek^4^ and resulted in no skin damage or ecchymosis. The benefit of adjusting the tension of the nasal SMAS at this region was weighed against the possibility of skin damage and long-lasting ecchymosis.

The third plane of dissection was the subperiosteal plane, which is the same as the subperiosteal plane of Cakir et al^3^. It allowed masking of any irregularity resulted from rasping and osteotomy and improves bone healing. This differed from the subcutaneous plane of Safe and Sadek's^4^ study. It was proved by Elshahat et al's^7^ study that the periosteum helps bone healing by acting as a natural barrier against soft tissue invasion.

The conclusion from this study was that the triple plane dissection collected the advantages of each single plane and avoided the disadvantages. The use of this described triple plane dissection technique is recommended for open primary rhinoplasty in Middle Eastern noses.

## Figures and Tables

**Figure 2:**
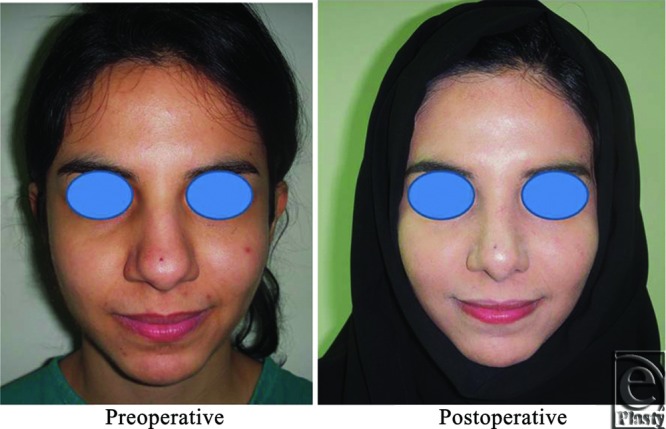
A front view photograph for a 24-year-old female patient shows the shape of the nose before and after open primary rhinoplasty. Skin thickness was N + 2 and defatting was performed.

**Figure 3:**
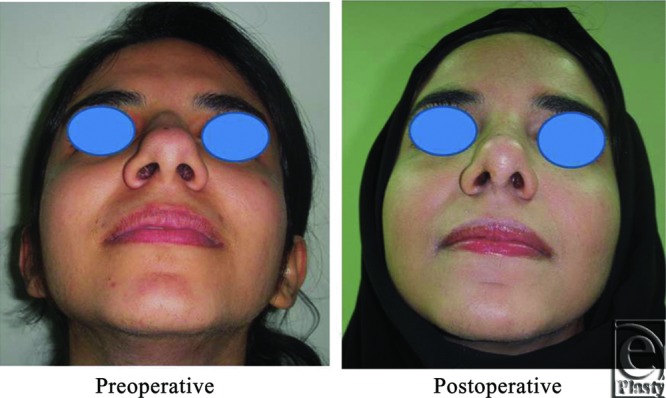
Base view photograph for the same patient in Fig 2 before and after open primary rhinoplasty.

**Figure 4:**
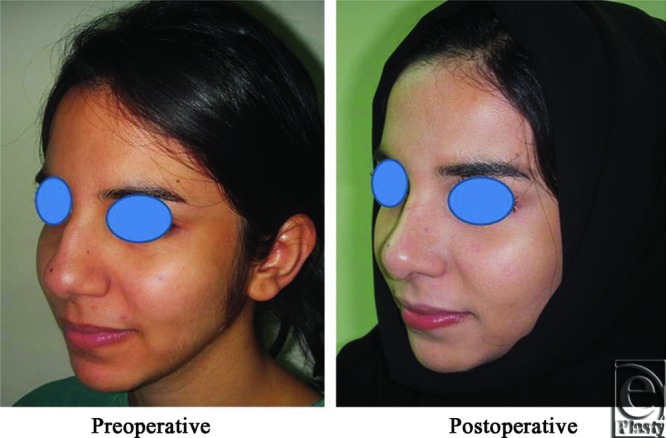
Left oblique view photo for the same patient Fig 2 before and after open primary rhinoplasty.

**Figure 5:**
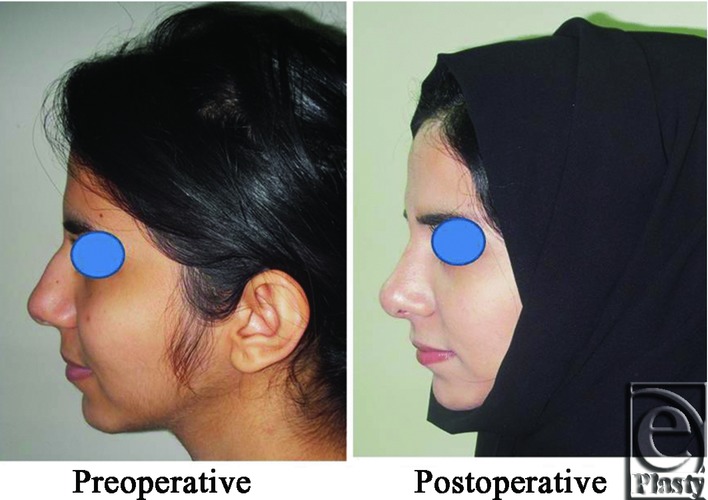
Left lateral view photograph for the same patient Fig 2 before and after open primary rhinoplasty.

**Figure 6:**
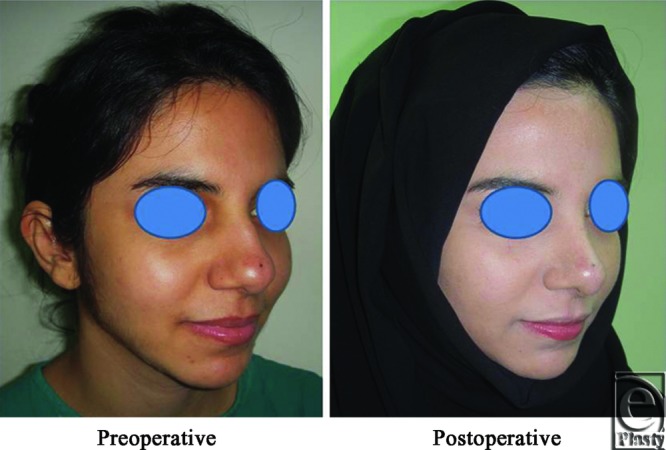
Right oblique view photograph for the same patient Fig 2 before and after open primary rhinoplasty.

**Figure 7:**
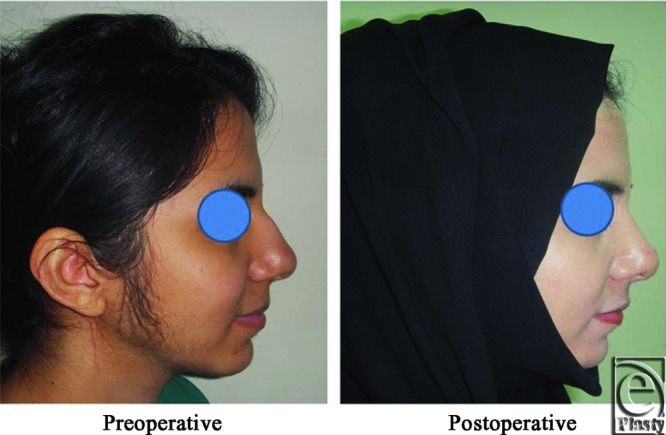
Right lateral view photograph for the same patient Fig 2 before and after open primary rhinoplasty.

**Figure 8:**
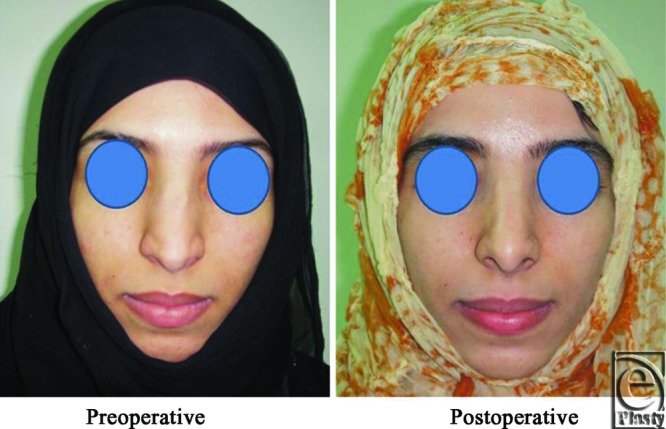
A front view photo for a 29-year-old female patient shows the shape of the nose before and after open primary rhinoplasty. Skin thickness was N + 1.

**Figure 9:**
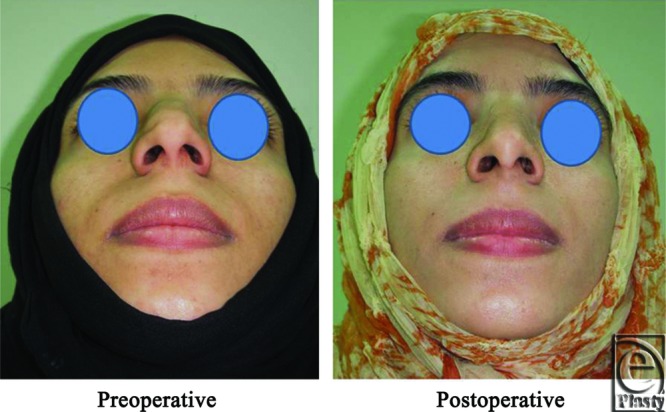
Base view photograph for the same patient in Fig 8 before and after open primary rhinoplasty.

**Figure 10:**
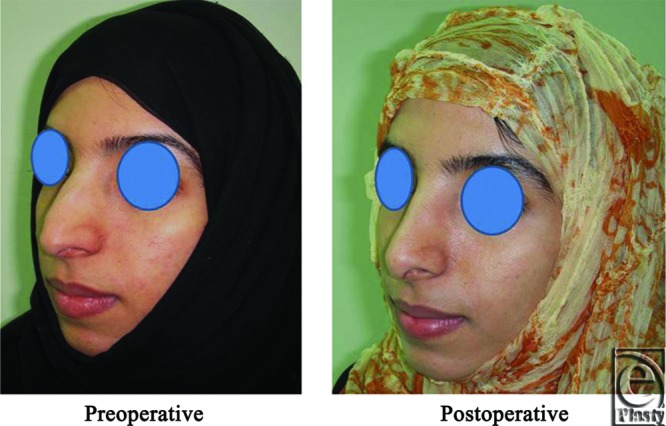
Left oblique view photograph for the same patient in Fig 8 before and after open primary rhinoplasty.

**Figure 11:**
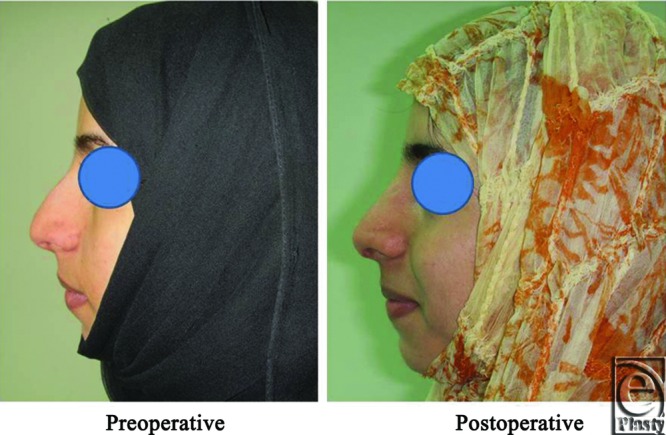
Left lateral view photograph for the same patient in Fig 8 before and after open primary rhinoplasty.

**Figure 12:**
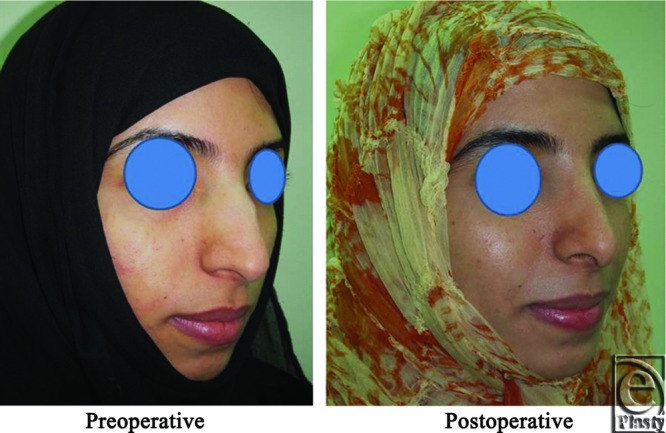
Right oblique view photograph for the same patient in Fig 8 before and after open primary rhinoplasty.

**Figure 13:**
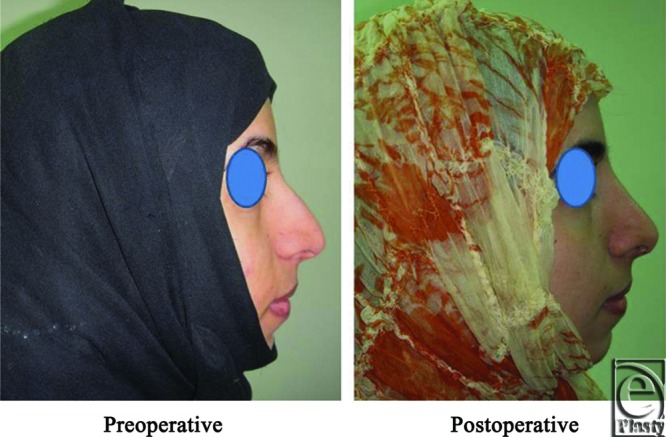
Right lateral view photograph for the same patient in Fig 8 before and after open primary rhinoplasty.

**Figure 14:**
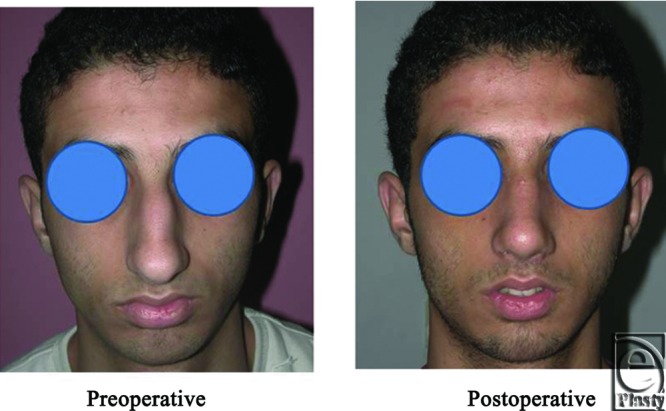
A front view photograph for a 27-year-old male patient shows the shape of the nose before and after open primary rhinoplasty. Skin thickness was N + 2 and defatting was performed.

**Figure 15:**
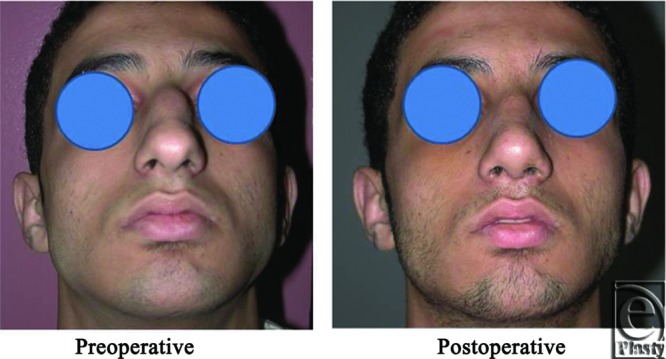
Base view photograph for the same patient in Fig 14 before and after open primary rhinoplasty.

**Figure 16:**
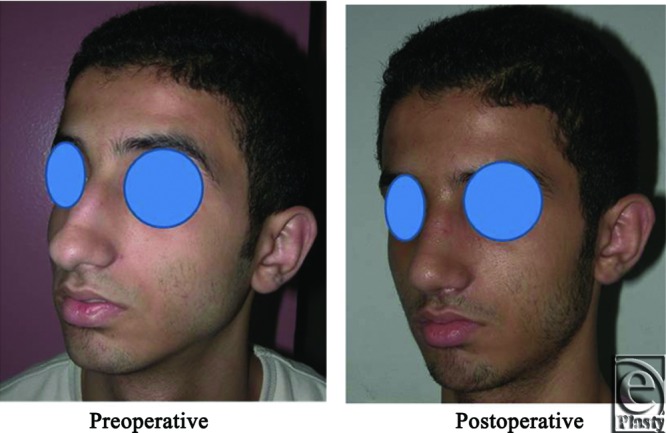
Left oblique view photograph for the same patient in Fig 14 before and after open primary rhinoplasty.

**Figure 17:**
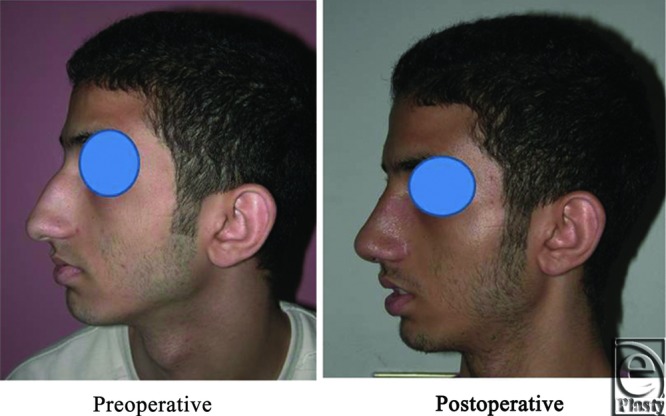
Left lateral view photograph for the same patient in Fig 14 before and after open primary rhinoplasty.

**Figure 18:**
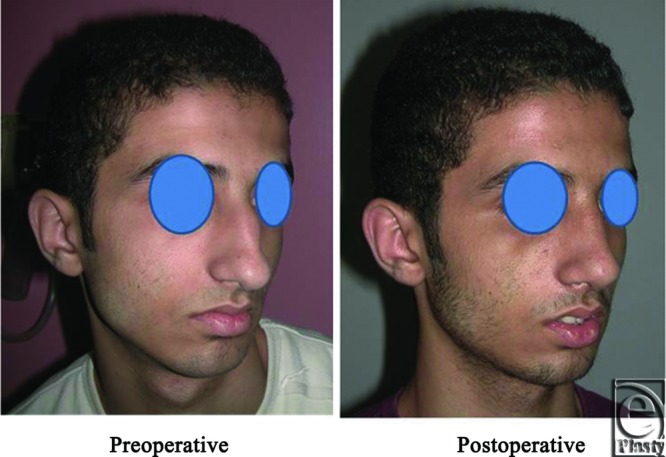
Right oblique view photograph for the same patient in Fig 14 before and after open primary rhinoplasty.

**Figure 19:**
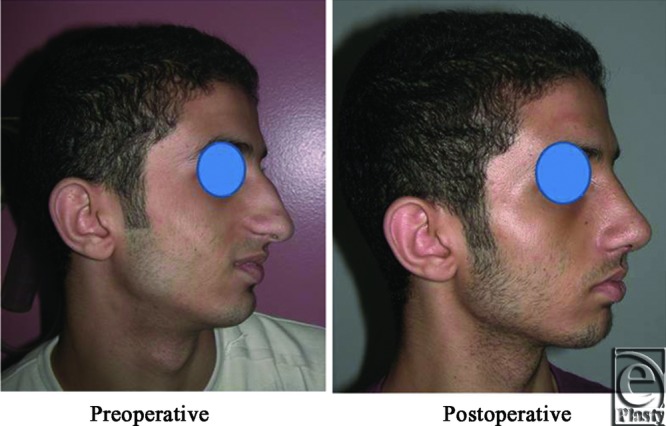
Right lateral view photograph for the same patient in Fig 14 before and after open primary rhinoplasty.

**Figure 20:**
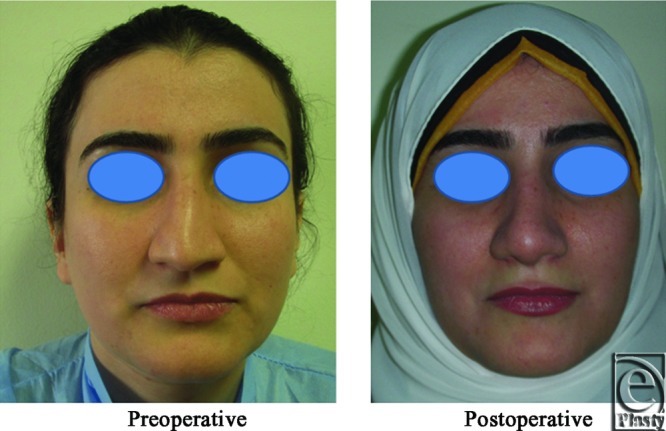
A front view photograph for a 31-year-old female patient shows the shape of the nose before and after open primary rhinoplasty. Skin thickness was N + 1.

**Figure 21:**
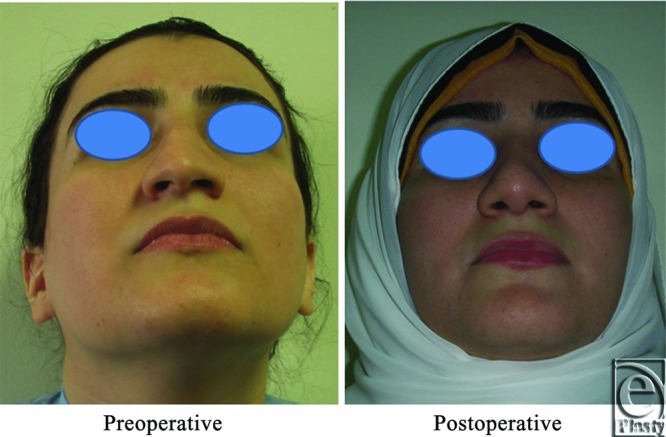
Base view photograph for the same patient in Fig 20 before and after open primary rhinoplasty.

**Figure 22:**
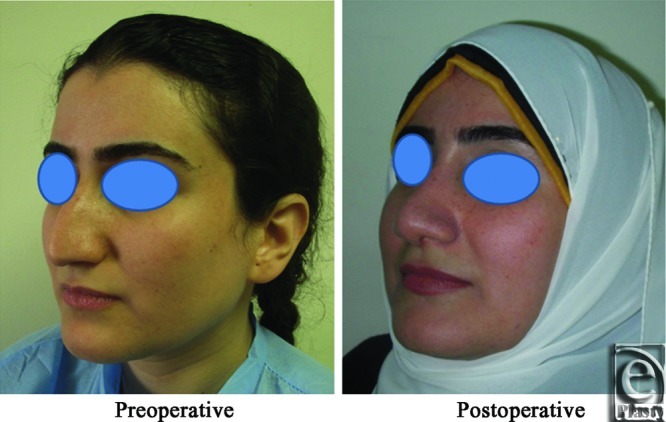
Left oblique view photograph for the same patient in Fig 20 before and after open primary rhinoplasty.

**Figure 23:**
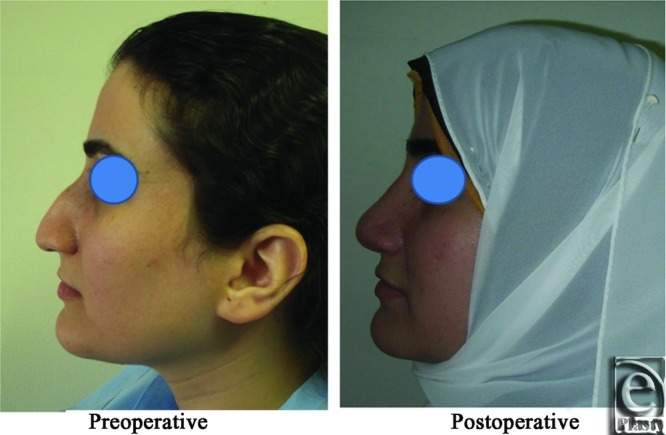
Left lateral view photograph for the same patient in Fig 20 before and after open primary rhinoplasty.

**Figure 24:**
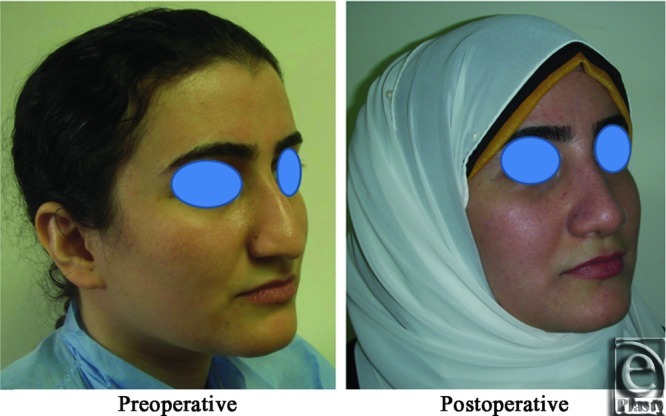
Right oblique view photograph for the same patient in Fig 20 before and after open primary rhinoplasty.

**Figure 25:**
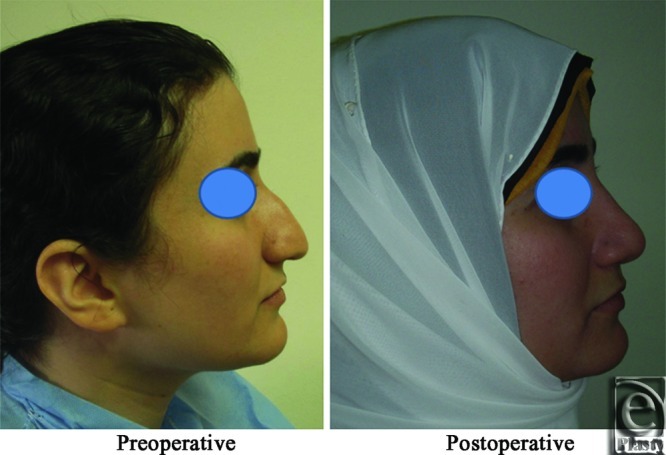
Right lateral view photograph for the same patient in Fig 20 before and after open primary rhinoplasty.

**Figure 26:**
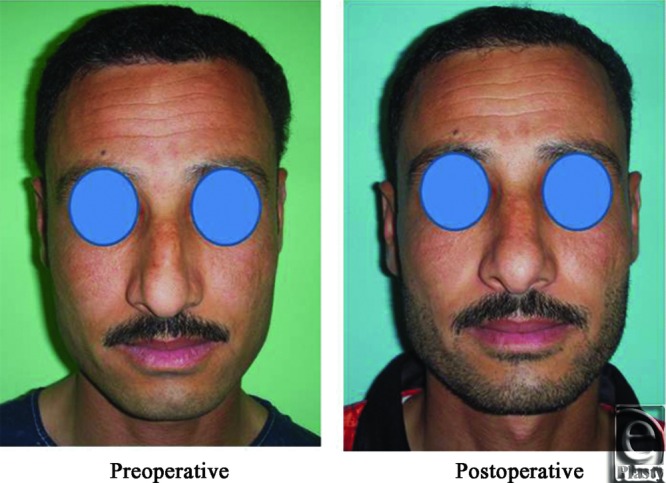
A front view photograph for a 37-year-old male patient shows the shape of the nose before and after open primary rhinoplasty. Skin thickness was N + 1.

**Figure 27:**
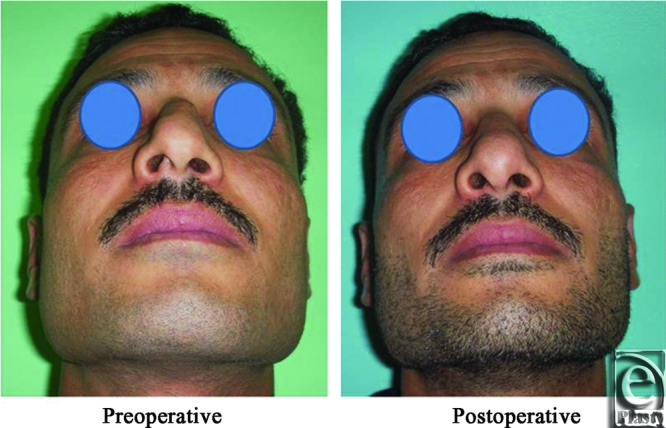
Base view photograph for the same patient in Fig 26 before and after open primary rhinoplasty.

**Figure 28:**
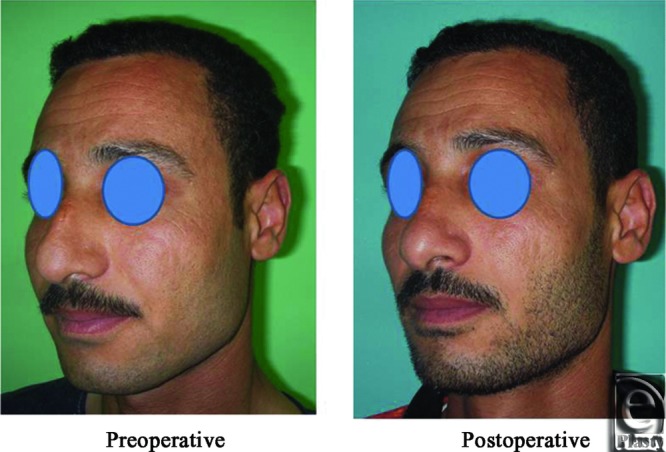
Left oblique view photograph for the same patient in Fig 26 before and after open primary rhinoplasty.

**Figure 29:**
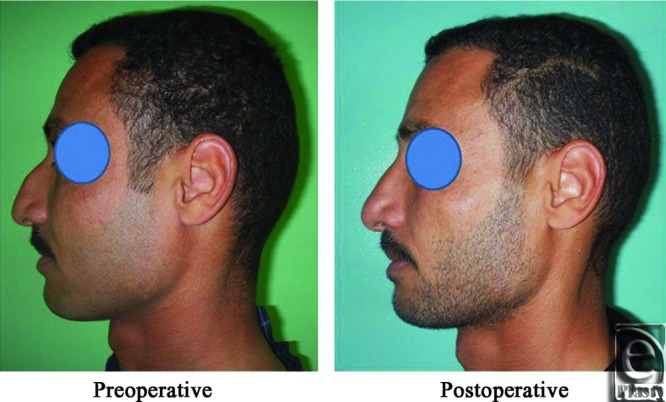
Left lateral view photograph for the same patient in Fig 26 before and after open primary rhinoplasty.

**Figure 30:**
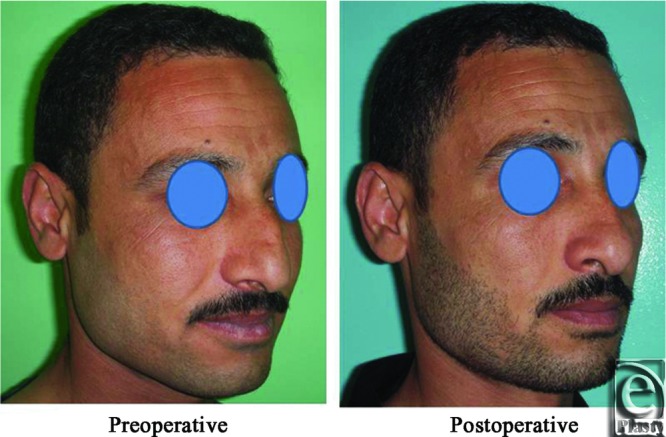
Right oblique view photograph for the same patient in Fig 26 before and after open primary rhinoplasty.

**Figure 31:**
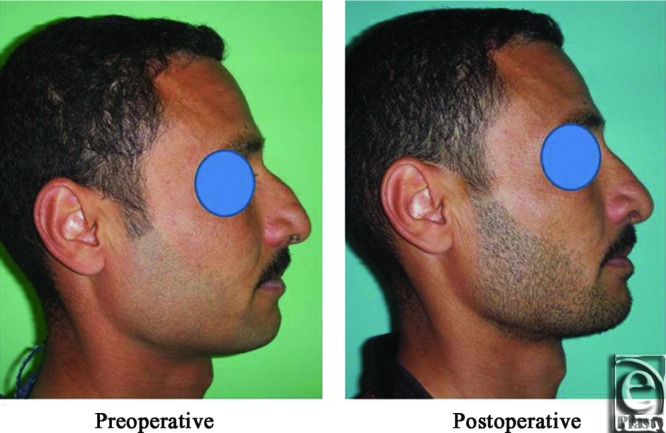
Right lateral view photograph for the same patient in Fig 26 before and after open primary rhinoplasty.

**Table 1:** Classification of patients based on skin thickness at the nasal tip as suggested by Daniel^5^ and the management of each group

Number of Patients	Classification	Description	Management
5	N + 3	Extraordinary thick skin	Extensive defatting
12	N + 2	Moderately thick skin	Moderate defatting
8	N + 1	Slightly thick skin	No defatting
10	N	Normal thickness skin	No defatting
3	N − 1	Slightly thin skin	Cautious elevation of skin from nasal SMAS over the alar cartilages
